# Genome-resolved metagenomics: a game changer for microbiome medicine

**DOI:** 10.1038/s12276-024-01262-7

**Published:** 2024-07-01

**Authors:** Nayeon Kim, Junyeong Ma, Wonjong Kim, Jungyeon Kim, Peter Belenky, Insuk Lee

**Affiliations:** 1 Department of Biotechnology, College of Life Science and Biotechnology, Yonsei University, Seoul, 03722 Republic of Korea; 2 Department of Molecular Microbiology and Immunology, Brown University, Providence, RI 02912 USA; 3 POSTECH Biotech Center, Pohang University of Science and Technology (POSTECH), Pohang, 37673 Republic of Korea

**Keywords:** Genome informatics, Genomics

## Abstract

Recent substantial evidence implicating commensal bacteria in human diseases has given rise to a new domain in biomedical research: microbiome medicine. This emerging field aims to understand and leverage the human microbiota and derivative molecules for disease prevention and treatment. Despite the complex and hierarchical organization of this ecosystem, most research over the years has relied on 16S amplicon sequencing, a legacy of bacterial phylogeny and taxonomy. Although advanced sequencing technologies have enabled cost-effective analysis of entire microbiota, translating the relatively short nucleotide information into the functional and taxonomic organization of the microbiome has posed challenges until recently. In the last decade, genome-resolved metagenomics, which aims to reconstruct microbial genomes directly from whole-metagenome sequencing data, has made significant strides and continues to unveil the mysteries of various human-associated microbial communities. There has been a rapid increase in the volume of whole metagenome sequencing data and in the compilation of novel metagenome-assembled genomes and protein sequences in public depositories. This review provides an overview of the capabilities and methods of genome-resolved metagenomics for studying the human microbiome, with a focus on investigating the prokaryotic microbiota of the human gut. Just as decoding the human genome and its variations marked the beginning of the genomic medicine era, unraveling the genomes of commensal microbes and their sequence variations is ushering us into the era of microbiome medicine. Genome-resolved metagenomics stands as a pivotal tool in this transition and can accelerate our journey toward achieving these scientific and medical milestones.

## Metagenomics: illuminating human gut microbiota for medical breakthrough

The human body houses numerous microbes, tiny organisms, that are vital for our health. This research aims to overcome limitations using genome-resolved metagenomics, a method that assembles complete genomes from complex microbial communities without needing to grow the organisms in a lab. The study focuses on the gut microbiome, using advanced computer methods to build metagenome-assembled genomes from DNA sequencing data. The research successfully increased the genetic diversity of the human gut microbiome by adding many new genomes to the existing database. The main findings include identifying new microbial species and expanding the genetic repertoire of known species, providing deeper understanding of the microbial diversity within the human gut. Researchers conclude that genome-resolved metagenomics is a significant advancement in microbiome research, offering understanding of microbial communities and their functions. This summary was initially drafted using artificial intelligence, then revised and fact-checked by the author.

## Introduction

The human body is home to a multitude of symbiotic microbial cells that outnumber the host’s own cells and exert a significant influence on human physiology. As evidence regarding the role of commensal microbes in human diseases has accumulated, microbiome medicine has emerged as a new field in biomedical research. This field seeks to harness human microbiota and derived molecules for the prevention and treatment of diseases. Achieving this goal requires a comprehensive understanding of the taxonomic and functional organization of the human microbiome.

Historically, microbial community research has been a domain within microbial ecology that was initially focused on environmental microbes. However, the discovery of vast microbial communities within the human body has expanded the scope of this field. For many years, human microbiome research has adopted methodologies based on bacterial phylogeny and taxonomy, particularly 16S rRNA gene sequence analysis^1^, which is adequate for revealing differences in taxonomic composition between diseased microbiomes and their healthy counterparts. However, the limited taxonomic resolution of 16S rRNA sequences^2^ and their inherent inability to perform functional analysis pose obstacles to further advancements, including identifying the functional elements of the microbiome that directly influence host physiology. This situation is reminiscent of human genetics prior to the availability of the human reference genome. The absence of a comprehensive human genome map meant that the search for disease genes was based on sparse genomic landmarks, leading to the identification of only broad chromosomal regions associated with diseases. This approach often requires years of subsequent studies to precisely locate the genes responsible. The decoding of the human genome and the cataloging of single nucleotide variations accelerated the discovery of disease-associated genes and genetic variations, thus ushering in the era of genomic medicine^3^.

In this review, we advocate for a similar transition in microbiome medicine. Decoding the complete genomes of all commensal microbial species and cataloging their genetic components will expedite the development of new biomarkers and therapeutics derived from the human microbiome. Genome assembly, especially for not-yet-cultured species, has been technically challenging for many years. However, recent advancements in *genome-resolved metagenomics* have ushered in significant changes in research. Numerous computational methods have been developed for de novo genome assembly from metagenome shotgun sequencing data, leading to the rapid accumulation of draft genomes in the form of *metagenome-assembled genomes* (MAGs). This review discusses computational methods for MAG construction and their impact on human microbiome research, with a particular focus on gut microbiome research. Additionally, while the same research framework can be applied to study various microbial communities in the body, this review primarily addresses the study of prokaryotic commensal microbes, noting that MAG reconstruction is also possible for symbiotic fungi and viruses.

## Inherent limitations of 16S rRNA gene sequencing

16S rRNA gene sequencing has been a popular method for taxonomic analysis of microbial communities due to its cost-effectiveness and straightforward bioinformatic interpretation, making it widely accessible. However, this approach has several inherent limitations related to its target of analysis, the 16S rRNA sequence.

First, the variation in 16S rRNA sequences generally does not permit taxonomic classification at the species level. Recent studies have shown that even the analysis of entire 16S regions using long-read sequencing might not be sufficient for species-level taxonomic differentiation^4^. Moreover, differences at the subspecies level between microbes of the same species can have significant impacts on host physiology, yet these nuances are often overlooked in taxonomic analyses of microbiomes. Second, 16S rRNA sequences do not provide information about the functional capabilities of microbes. Although tools such as PICRUSt^5^ allow for the prediction of metabolic pathways based on 16S rRNA sequences, the results are mere inferences drawn from a limited number of representative genomes associated with a given 16S rRNA sequence. Third, 16S rRNA sequences are unique to prokaryotes, rendering the detection of nonbacterial commensals, such as fungi, viruses, and protists, impossible using this sequence information. Fourth and most critically, the study of novel species that are considered ‘microbial dark matter’ is challenging, as the interpretation of 16S rRNA sequences is heavily reliant on databases populated with known bacterial species. This dependency can impede the discovery and understanding of previously uncharacterized microbial entities.

## Microbiome analysis with whole-metagenome sequencing (WMS): a new paradigm

The Human Microbiome Project (HMP)^6^ differed from the Human Genome Project in that it did not produce reference genomes from sequencing data. This was due to the complexity of assembling individual bacterial genomes from mixed sequence reads originating from various bacterial sources. At that time, computational algorithms were not advanced enough to effectively separate and assemble these genomes accurately. Nonetheless, HMP was crucial in shifting microbiome research toward WMS, which involves sequencing all genetic material in a sample to provide a more comprehensive understanding of the microbiome.

**Fig. 1:**
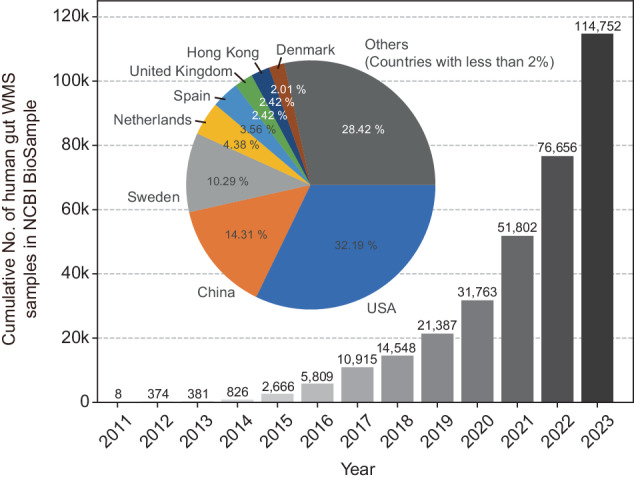
Distribution of human gut whole metagenome sequencing (WMS) samples submitted to the NCBI Sequence Read Archive (SRA) by country and year. The bar graph shows the annual cumulative number of human gut WMS samples submitted to the NCBI SRA. The pie chart inset breaks down the contribution of different countries to the total sample submissions; the USA contributed the majority, followed by China, Sweden, and other countries as of the last recorded year. Countries contributing less than 2% are grouped under “Others.” This figure highlights the increasing growth rate and geographical bias of human gut WMS data in public databases.

HMP significantly contributed to human microbiome research by releasing WMS datasets from the healthy human microbiome to the public. These datasets included 541 samples from the gut, 215 from the vaginal microbiome, 1090 from the oral microbiome, and 56 from the skin microbiome, highlighting the project’s broad scope and its impact on understanding human health. This release led to the development of numerous bioinformatics tools for analysis. The second phase of the HMP, known as HMP2 or iHMP, aimed to provide a more comprehensive understanding of host-microbiome interactions over time^7^. Extensive multiomics data encompassing host and microbiome interactions were generated for HMP2. These included WMS data related to the human gut and vaginal microbiome during pregnancy and preterm birth, inflammatory bowel diseases, and prediabetes. As a result, the public database was enriched with WMS data from an additional 2000 gut samples and 930 vaginal samples, thus further advancing the resources available for human microbiome research. Thanks to this large-scale consortium project and numerous other studies, the volume of public WMS datasets for the human gut microbiome has grown rapidly, exceeding 110,000 samples by 2023 (Fig. 1). However, a notable issue is the significant geographical bias in the data. Most of the public WMS data originate from a few countries, such as the US, China, and some European nations, leaving gut microbiome data from most Asian and African countries underrepresented. This gap is critical, as the gut microbiota composition is heavily influenced by diet and lifestyle^8–10^. The current human gut microbiome data landscape, therefore, lacks comprehensiveness. Addressing this imbalance by including underrepresented populations in future sample collections and analyses is essential for a more accurate global understanding of the human gut microbiome.

## Genome-resolved metagenomics: enabling versatile study of the human microbiome

**Fig. 2:**
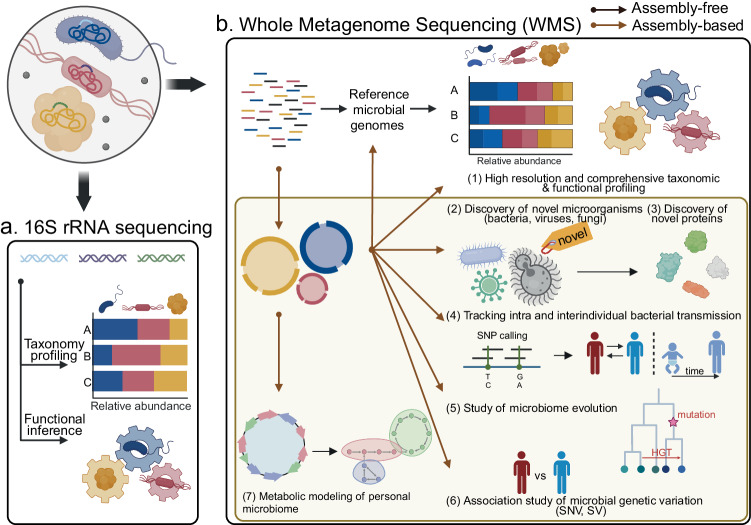
Comparison of 16S rRNA sequencing and whole-metagenome sequencing (WMS) in microbiome analysis. **a** 16S rRNA sequencing analysis can be used to perform taxonomy profiling and functional inference based on the taxonomic profile. **b** Various routes of microbiome analysis through the WMS, which include both assembly-free and assembly-based approaches. The figure emphasizes the comprehensive insights provided by WMS in understanding microbiomes compared to 16S rRNA sequencing.

Genome-resolved metagenomics is a transformative approach in microbiome studies, delving into the DNA of mixed microbial communities to directly assemble and analyze individual genomes from metagenomic data. This technique marks a significant advancement over traditional 16S rRNA sequencing, offering an enriched depth of understanding and unprecedented insights into the human microbiome (Fig. 2).

At the core of this method, genome-resolved metagenomics allows for the assembly of novel genomes spanning a variety of microorganisms, encompassing bacteria, viruses, and fungi. Including these novel species genomes extends the phylogenetic tree, thus bringing previously undetectable species into focus^11^. Furthermore, the increasing availability of genomic data at the species level facilitates in-depth investigations of variations within species^12^. This advancement lays the groundwork for the development of comprehensive pangenomes^13^, which would offer a more detailed understanding of the genetic diversity within species. Researchers are now poised to uncover numerous novel coding sequences, which could lead to the identification of new metagenome protein families^14,15^. Genomic comparisons within bacterial species facilitate the tracking of the intra- and interindividual transmission of commensal bacteria^16,17^, while genome-centric analysis offers a window into microbiome evolution through genetic mutations and horizontal gene transfer^18^. The within-species genetic diversity reflects the microbiome’s adaptive journey within specific host environments^19^, thus revealing potential statistical associations between single nucleotide variants (SNVs) or structural variants (SVs) of microbial genomes and host phenotypes^20,21^. Finally, MAGs enable us to conduct genome-scale metabolic modeling for uncultured bacterial species^22^, representing a substantial portion of the human gut microbiome, ultimately allowing for the metabolic modeling of individual microbiomes^23^.

## Assembly of individual microbial genomes from metagenomic sequencing reads

**Fig. 3:**
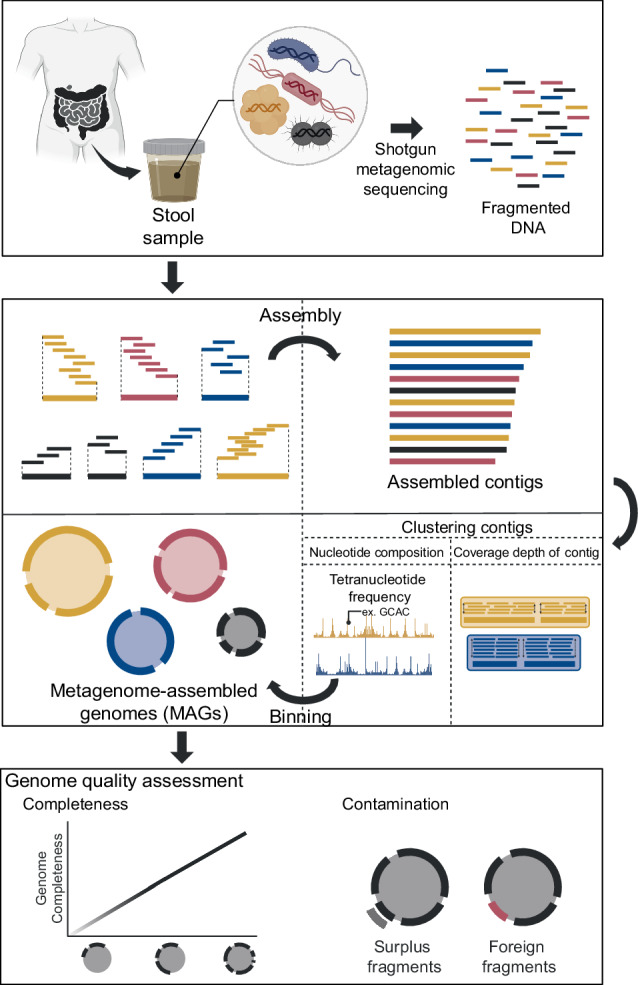
Workflow of metagenome-assembled genome (MAG) reconstruction from metagenomic samples. This flowchart outlines the process of generating MAGs from a stool sample. The procedure begins with the collection of a stool sample, followed by shotgun metagenomic sequencing to obtain fragmented DNA. The DNA fragments are then assembled into contigs. These contigs are clustered based on nucleotide composition and coverage depth to form MAGs through the binning process. The final step involves a quality assessment of the assembled genomes, evaluating completeness and checking for contamination.

Generating MAGs from mixed short-read sequences originating from various microorganisms is the first step in genome-resolved metagenomics. The construction of MAGs comprises a two-step process that includes assembly and binning (Fig. 3).

During the initial assembly step, short reads are pieced together into longer contigs, resembling the assembly of a puzzle, where the overlapping regions of these short reads serve as the connecting elements. Generally, there are two assembly models: the overlap-layout-consensus (OLC) model and the *De Bruijn* graph. In the OLC model, each read is represented as a node in a graph, with the overlaps between reads depicted as edges. However, as sequencing depth increases, this method can lead to large and complex graphs. Conversely, the *De Bruijn* graph model enhances scalability by dividing reads into k-mers^24^. Short-read assemblers such as metaSPAdes^25^ and MEGAHIT^26^ employ this strategy by splitting short reads into k-mer fragments and then using *De Bruijn* graphs to assemble these fragments into extended contigs^27^. The assembly process can be undertaken in two ways: single-assembly, which is performed independently for each sample, and coassembly, which is carried out on merged samples after pooling multiple samples^28,29^. Each method has distinct advantages and drawbacks (Supplementary Table 1a). Unlike environmental samples, such as those from interconnected ocean and soil, the human gut microbiome represents a distinct environment that varies among individuals. Consequently, preserving strain-specific variants such as SNVs is crucial. The preservation of strain specificity is attainable through diverse paths in the *De Bruijn* graph. However, this process results in the generation of numerous fragmented contigs^30,31^. Therefore, we recommend employing a single-assembly approach. If the goal is to capture low-abundance taxa, increasing sequencing depth instead of coassembly is advisable^32^.

Moving on to the binning step, contigs originating from the same genome are grouped into bins, each corresponding to a specific genome. Binning involves clustering similar contigs based on their sequence composition and coverage depth^33–36^. The sequence composition refers to nucleotide features, including k-mers. Given that a species is distinguished by the constancy of k-mers and GC ratios throughout its genome^37–39^, these features can be employed to cluster contigs into a genome bin. The tetranucleotide frequency (TNF) is the most frequently utilized metric for this purpose and has demonstrated superior performance in comparison to other k-mer sizes^36^. Additionally, contigs from the same genome are co-abundant in a sample^40^, making contigs with comparable coverage depths more likely candidates for belonging to the same genome. The coverage depth can be calculated from a single sample (single-coverage binning) and from a group of samples (multi-coverage binning)^41^. These two approaches present advantages and disadvantages (Supplementary Table 1b). Single-coverage binning based on co-abundance within a single sample may inadvertently introduce contaminated contigs into a genome bin, which can affect downstream analyses. To mitigate this issue, we suggest adopting multi-coverage binning using co-abundance across multiple samples. Implementing this approach requires careful consideration of which samples to collectively analyze in multi-coverage binning to ensure accuracy and reduce the risks of contamination.

Furthermore, in the clustering of contigs from the same species, various tools differ in the features and algorithms employed for binning (Supplementary Table 1c)^33–35,42–47^. Given that no single tool universally outperforms in all scenarios^48,49^, using several binning tools and combining their results through ensemble methods is common. The merging step, which is referred to as bin refinement, combines the results of multiple binning tools to create a single bin with the highest quality combination of contigs^50^. The tools used for this process are summarized in Supplementary Table 1d^51–53^.

As the generated genome sequences can be used in various downstream analyses, we need to measure the quality of the final bin, i.e., a single genome sequence. While there are quantitative quality metrics such as the N50 and number of contigs, there are two absolute metrics for measuring genome quality that universally define MAG quality: completeness and contamination^54,55^. The reliability of a genome sequence is directly proportional to its completeness and inversely proportional to its contamination level. According to the widely recognized Minimum Information about a Metagenome-Assembled Genome (MIMAG) standard^56^, a genome with over 50% completeness and less than 10% contamination is classified as a medium-quality draft genome. In contrast, a genome with more than 90% completeness and less than 5% contamination is considered a near-complete draft genome. Completeness refers to how much of the actual genome is covered by the assembled genome sequence. Low completeness of a genome sequence can result in an underestimation of the functional capacity of a species when inferring its functional capabilities or conducting metabolic modeling^57^. Contamination in a genome sequence indicates the presence of extraneous fragments that do not belong to the genome being sequenced^58^. Contamination in genome sequences arises from various sources, including the mixing of closely related genomes during the binning process due to their similar sequence compositions. Additionally, genomes that are taxonomically distant can become contaminated for various reasons. Various computational tools are available to detect contamination in a genome (Supplementary Table 1e)^54,59^. For comprehensive quality control, using multiple tools due to their differing strengths is advisable^60,61^. Another common source of contamination is the inclusion of sequences from the host, such as human DNA in microbiome studies, or that from fungal and viral sequences. Particularly for this third type of contamination that involves eukaryotic or viral sequences, extra caution is necessary.

## Expansion of phylogeny through MAGs and their taxonomic classification

Recent advancements in bioinformatics and the reduced cost of metagenomic sequencing have greatly facilitated the large-scale construction of bacterial MAGs, which require accurate taxonomic classification. Traditionally, the classification of bacterial genomes has relied on the National Center for Biotechnology Information (NCBI) taxonomy, a system grounded in the International Code of Nomenclature of Prokaryotes^62^. However, this consensus-based nomenclature system often struggles to keep up with the swift identification and categorization of new species. To address these challenges, an automatic and objective approach for classifying new bacterial and archaeal genomes involves their integration into a reference phylogenetic tree. The Genome Taxonomy Database (GTDB)^63^, a reference bacterial taxonomy database, provides a contemporary solution for this. Unlike the NCBI taxonomy, which often uses the 16S rRNA region for classification, GTDB bases its reference on 120 specific single-copy marker proteins for bacterial genomes. GTDB has also made efforts to rectify common issues in traditional taxonomy, such as the removal of polyphyletic groups to align phylogeny with taxonomy^64^ and the normalization of unequal taxonomic ranks^65^. The GTDB Toolkit (GTDB-Tk)^66^ was developed to facilitate the accurate taxonomic classification of novel genomes by placing them within the GTDB framework. This phylogenetic reference allows for the genome sequence-based taxonomy annotation of new species by determining their phylogenetic positions. While most the species in the GTDB currently carry nonstandard placeholder names, this system allows for taxonomy annotation based on genome sequences, even for novel species, by inferring their position in the phylogeny.

**Fig. 4:**
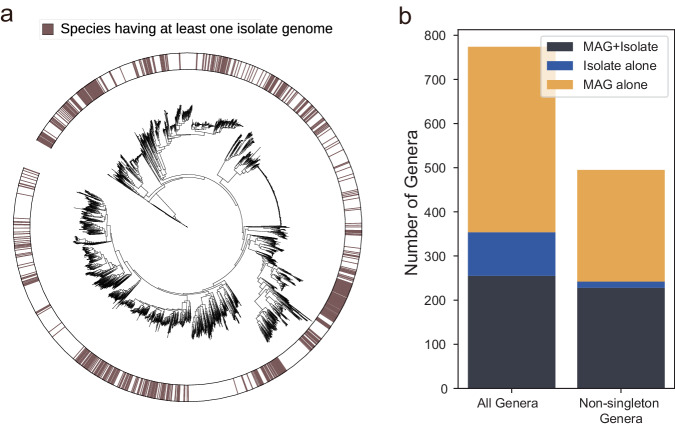
Comparison of species or genera having isolate genomes and metagenome-assembled genomes (MAGs) and those with MAG alone. **a** The phylogenetic tree represents 5414 microbial species cataloged in the Human Reference Gut Microbiome (HRGM), 893 of which (16.5%) possessed at least one isolate genome marked on the outer ring. **b** Bar chart comparing the number of genera composed of isolate genomes alone, MAGs alone, and those with both isolate genomes and MAGs. The ‘Non-singleton Genera’ column shows numbers excluding genera represented by a single species. This visualization underscores the extent to which MAGs complement isolate genomes in representing microbial diversity, particularly within non-singleton genera.

Many MAGs have revealed novel microbial species, thereby significantly expanding the current phylogenetic tree. This advancement is particularly evident in the study of the human gut microbiome, where a limited number of species have been isolated, leaving the vast majority uncultured^67^. For instance, to date, fewer than 20% of the prokaryotic species cataloged in the Human Reference Gut Microbiome (HRGM)^32^ have at least one genome assembled from an isolated strain (isolate genome), with the majority of species being defined solely by MAGs (Fig. 4). Notably, several large clades of bacterial taxa have not yet had any isolate genome. With the increasing ease of assembling genomes for uncultured species via MAGs, phylogenetic trees representing prokaryotic life are poised for rapid expansion^63,68^.

## Functional annotations of MAGs: making sense of novel genomes

Compiling a part list of a genome is a fundamental step toward understanding bacterial species, involving the prediction of open reading frames (ORFs) in MAGs and the annotation of their functions. A recent benchmark study assessed various gene prediction tools, revealing no universally superior tool^69^. However, Prodigal^70^, widely recognized as the most popular tool in this domain, consistently showed robust performance in different scenarios. Prodigal identifies key features in sequences, such as the ribosome binding site (RBS) motif, start codon usage, and coding statistics, through unsupervised learning. This approach allows for efficient gene prediction in non-model bacterial organisms. Once ORFs are predicted, they undergo automated functional annotation, where each gene is assigned, functional terms based on its homology to known proteins. A widely used database for such homology-based functional annotation is eggNOG^71^, which contains millions of orthologous groups (OGs). Compared with other annotation tools, eggNOG-mapper^72^ is particularly notable for its high accuracy, which can be achieved by effectively distinguishing between paralogs—similar sequences with potentially different functions^73^. Other tools offering homology-based functional annotations include InterProScan^74^, Prokka^75^, Bakta^76^, DRAM^77^, and MicrobeAnnotator^78^. Functional annotation tools leverage a range of databases to provide annotations, prominently including KEGG (pathway/orthology)^79^, Gene Ontology^80^, Pfam^81^, and Carbohydrate-Active Enzyme Database (CAZy) annotations^82^. These databases are widely used for their comprehensive resources in various domains of biological research.

Genome mining is a crucial method for identifying genes with specialized functions in prokaryotic genomes, particularly in the context of antibiotic resistance, which is a growing concern for human health due to the widespread use of antibiotics. This excessive use has led to the emergence of antibiotic-resistant pathogens^83^. Antibiotic resistance genes (ARGs), which may originate in commensal bacteria, become a significant risk when they are transferred to pathogens via horizontal gene transfer (HGT)^84–86^. Recent studies on the human gut microbiome have shown a link between antibiotic consumption and the prevalence of ARGs in the microbiome^87–89^. The detection of ARGs typically involves aligning sequences with a known ARG reference database for homology-based identification using tools such as RGI^90^ or ABRicate^91^. For identifying novel ARGs, methods such as the hidden Markov model (HMM) approach, exemplified by ResFam^92^ or fARGene^93^, and deep learning techniques such as deepARG^94^ or PLM-ARG^95^ are also employed. These methods rely on a database of known ARG sequences for accurate identification. Integrating multiple databases, including CARD^90^, ResFinderFG^96^, and MEGARes^97^, to enhance the comprehensiveness and accuracy of ARG detection is common.

Antimicrobial peptides (AMPs) are short peptides, typically composed of fewer than 100 amino acids^98^, that inhibit the growth of various microorganisms, including bacteria, fungi, parasites, and viruses^99^. These peptides are being evaluated as potential antibiotic alternatives, largely due to their anti-inflammatory and immunomodulatory properties^100^. Given the diversity of the human gut microbiome, it is expected to be a rich source of novel AMPs^101^. Research has increasingly focused on AMPs derived from the human gut microbiome, as they are likely nontoxic to human cells^102^. Machine learning-based methods have proven more effective than homology-based methods in identifying AMPs, owing to their short length^103,104^. Furthermore, recent efforts have been directed toward using deep learning techniques for the discovery of novel AMP candidates within the human gut microbiome^102,104,105^.

## Expansion of the pangenome through MAGs: uncovering the full functional potential of individual microbial species

**Fig. 5:**
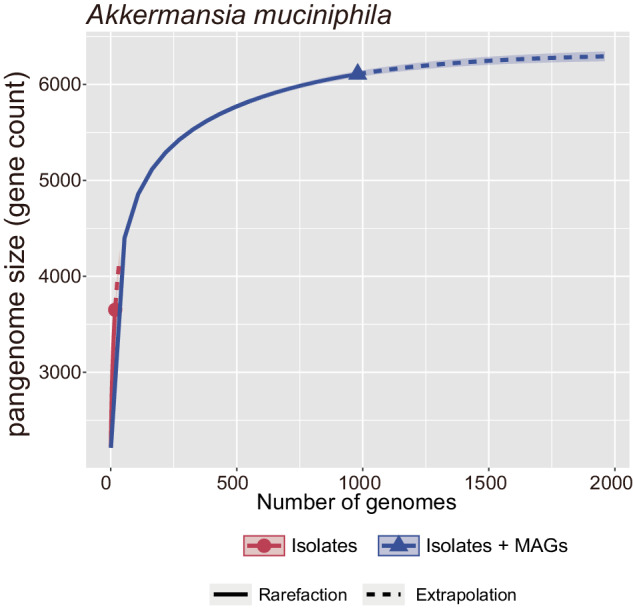
Expansion of the *Akkermansia muciniphila* pangenome with isolates and metagenome-assembled genomes (MAGs). This graph depicts the growth of the pangenome size (gene count) of *Akkermansia muciniphila* as more genomes are sequenced. The solid line indicates the rarefaction curve for isolate genomes only, showing initial rapid growth in pangenome size that begins to plateau as the number of genomes increases. The dashed line represents the extrapolation curve when both isolates and MAGs are considered, suggesting a larger pangenome. This illustrates the impact of incorporating MAGs on understanding the genomic diversity of this species, highlighting that MAGs substantially increase the known gene repertoire beyond what is observed with isolates alone.

The collection of MAGs for individual species provides insight into their functional potential, often conceptualized as a pangenome, which encompasses all the genes within a species, including both a core genome, comprising genes common to most strains, and an accessory genome, made up of genes found in only a subset of strains^106,107^. The inclusion of MAGs has led to a significant expansion in pangenome size for many species, surpassing what is observed with pangenomes constructed solely from isolate genomes. This phenomenon is exemplified by the pangenome analysis of *Akkermansia muciniphila* in the HRGM (Fig. 5). The accessory genome, which contributes to functional diversity within subspecies^108^, plays a crucial role in adaptation to different hosts and can be associated with pathogenic traits^109,110^. Thus, developing a comprehensive pangenome that distinguishes between core and accessory genomes is vital for a deeper understanding of the diversity within microbial species across different host populations.

The basic method for conducting pangenome analysis involves collecting all protein sequences from a species and clustering them to identify homologous genes^111^. Although this approach is efficient, it does not differentiate between paralogs, which are genes derived from gene duplications within the genome that often evolve distinct functions^112^. A more advanced technique groups homologous genes while preserving synteny using graph-based methods that incorporate information about gene neighborhoods^113^. This strategy is widely used in pangenome analysis tools such as Roary^114^, PPanGGOLiN^115^, and panaroo^116^. However, these tools can encounter difficulties in accurately grouping genes with weak synteny conservation, particularly those affected by HGT between different species^111^.

The quality of genomes plays a critical role in pangenome analysis. MAGs, while convenient to obtain, often face quality issues such as fragmented assemblies and potential contamination during the binning process. Fragmented assemblies can lead to gene loss, particularly at contig ends, which may affect the core genome. Contamination, conversely, might result in false positives within the accessory genome, leading to apparent expansion^117^. Panaroo is a widely used tool for pangenome analysis with MAGs, adept at managing challenges associated with MAGs, such as truncated ends and potential contamination, despite not being specifically designed for MAGs. Recently, ggcaller^118^, another tool, has been introduced to address similar challenges in pangenome analysis.

## Cataloging microbial genomes for specific environments: toward a comprehensive reference microbiome

Taxonomic and functional profiling of microbiome samples can be achieved through either assembly-based or assembly-free approaches. The assembly-based method involves de novo assembly of sequence reads into contigs and species bins, which, while not reliant on reference microbial genomes, is time-consuming and computationally intensive. Consequently, for commonly studied environments such as the human gut, the preferred method is the assembly-free approach that utilizes reference microbial genomes. The extensive collection of MAGs has prompted in-depth research into the creation of biome-specific reference databases designed for specific environments^119^. These references provide a rich source of genome and protein sequences for a given environment to aid in the identification of previously unknown genomes and proteins. Using biome-specific references as databases is especially advantageous for studying complex environments such as the human gut microbiome, facilitating quick and precise taxonomic and functional profiling of metagenomic samples without the need for de novo assembly.

There are several distinct catalogs of reference microbial genomes specific for the human gut. The Unified Human Gastrointestinal Genome (UHGG)^120^ is a comprehensive catalog that merges three prior large-scale collections of human gut bacterial genomes^11,61,67^. The UHGG provides 204,938 nonredundant genomes across 4644 prokaryotic species. However, the UHGG collection exhibits a geographical bias, primarily representing samples from the US, China, Denmark, and Spain, and thus lacks representation of gut microbes from various other regions. To mitigate this limitation, the HRGM^32^, in which MAGs from fecal metagenome samples from three East Asian countries—Korea, India, and Japan—were added, was introduced. The HRGM expands the range to 232,098 nonredundant genomes across 5414 prokaryotic species, increasing both genome and species numbers by approximately 10% compared to those of UHGG. These catalogs have markedly enhanced the classification of taxonomic reads beyond what is available in traditional catalogs such as the Reference Sequence (RefSeq) database, thus emphasizing the significance of having comprehensive catalogs for studying the human gut microbiome^32,120^. Further efforts have produced additional gut microbiome catalogs focusing on underrepresented geographic areas such as Israel^121^, Singapore^122^, and Inner Mongolia^123^. Additionally, cataloging MAGs from the fecal metagenomes of children under three years old has revealed many new microbial species, offering valuable insights for the study of the human gut microbiome in early life^61^.

## Sequence-resolved microbiome analysis: a population genetics perspective on the human microbiome

The key benefit of genome-resolved microbiome analysis lies in the application of genomics to study the human microbiome. With access to numerous genomes across various subspecies, exploring the genetic diversity within species of the human microbiome has become possible^12^. This genetic variation is crucial for several applications, including tracking identical strains, identifying links between specific strains and host phenotypes, and discovering bacterial genetic variants that correlate with host phenotypes^124^. Strain-level profiling, which utilizes single nucleotide variants (SNVs) among strains, has been instrumental^125^. Recent research has demonstrated the association of bacterial SNVs with host phenotypes, such as body mass index, underscoring the importance of nucleotide-level diversity in microbiome research^21^. Efforts to profile bacterial structural variants (SVs) in the human microbiome have also been made by examining their relationships with human health^20^. For instance, SVs in human gut bacteria have been linked to bile acid metabolism^126^ and the response to immune checkpoint inhibitors^127^, thus highlighting the significant insights genome-resolved analysis offers in understanding the human microbiome.

The traditional method for detecting bacterial genetic variations involves culturing microorganisms, isolating their genomes, sequencing them, and then identifying mismatches through whole-genome alignment^128^. However, this approach is less effective for the human gut microbiome, as a significant portion of the microbiome remains uncultured. An alternative strategy, metagenotyping, involves aligning WMS reads against reference microbial genomes to identify genetic variations; this strategy offers a feasible solution for analyzing the human gut microbiome. Key tools for read alignment-based metagenotyping include StrainPhlAn^129^, metaSNV^130^, and MIDAS^131^. These methods, while comprehensive, are time intensive and require high read coverage to accurately differentiate between actual genetic variations and sequencing errors. To overcome these limitations, newer tools such as GT-Pro^132^ that employ exact k-mer matching algorithms for metagenotyping have been developed. K-mer-based methods are quicker than read alignment-based techniques, although they may sometimes be less accurate.

Metagenotyping has become a key tool in exploring the transmission of microbial communities at the strain level. The applications of metagenotyping range from studying microbe transfer within the body, such as from the oral cavity to the gut, to investigating how specific diseases may be linked to oral-to-gut microbial transmission^17,133^. In addition to individual-level studies, metagenotyping is instrumental for examining interindividual microbial transfers, including vertical transmission from mothers to infants and microbial sharing within households or larger populations^16,134^. Another significant application of metagenotyping is analyzing strain-level changes in the gut microbiome composition following fecal microbiota transplantation (FMT), providing valuable insights into this therapeutic intervention^135–137^. These examples underline the versatility and potential usefulness of metagenotyping in various transmission-related research areas.

Metagenotyping has also proven effective in tracking the evolutionary dynamics of gut microbiomes, both within individuals and across different individuals. When applied to longitudinal samples from a single person, this technique allows for comparisons of strain similarities within and between individuals. Metagenotyping also enables the observation of how specific strains of species evolve over time within an individual^129,138–141^. Additionally, metagenotyping has been useful for detecting genetic changes in gut microbes that occur in response to external influences, such as antibiotic treatments^142^. These applications suggest that metagenotyping holds considerable potential for use in broader studies, particularly in examining how various factors induce genetic variations in gut microbes.

## Metabolic modeling of MAGs: enabling metabolic simulation of personal microbiomes

As personalized medicine advances, simulating host-microbiome metabolic interactions is becoming essential for forecasting health outcomes and customizing treatments^143–145^. In the past, the field of metabolic engineering primarily used genome-scale metabolic models (GEMs) that were reconstructed for culturable species with complete genome sequences to predict genetic content^146,147^. Currently, the recent surge in the availability of numerous MAGs has opened the door to reconstructing GEMs for gut commensal microbes that are not yet culturable.

The main objective in reconstructing GEMs is to chart the behaviors of specific organisms and predict their interactions within individual models. The reconstruction of genome sequences is a meticulous and laborious process that requires thorough curation. Given the immense diversity of microbes in the human microbiome, which encompasses thousands of species, automation of this process is vital. To this end, several tools have been developed for automated GEM reconstruction, such as RAVEN^148^, Pathway Tools^149^, and merlin^150^, which greatly aid in MAG-based metabolic modeling. Notable among these tools are ModelSEED^151,152^, CarveMe^153^, and gapseq^154^. Generated GEMs can be evaluated using MEMOTE^155^, which offers a standardized method for quality assessment. This tool ensures that GEMs meet specific criteria for accuracy and completeness, thus facilitating their use in research and application.

ModelSEED, a web-based platform, streamlines the process of generating draft metabolic models. This platform utilizes the SEED framework pipeline, which begins with assembling genome sequences and submitting them to the RAST annotation server for genetic content prediction^156^. This process involves constructing gene‒protein-reaction associations, generating biomass reactions, assembling the reaction network, and analyzing reaction reversibility thermodynamics. The end result is an optimized draft model. The AGORA project, using the ModelSEED pipeline, has produced over 7000 GEMs for human gut bacteria, combining automated draft model generation from MAGs with manual curation^157,158^.

CarveMe is a command-line tool designed for the quick, automated reconstruction of GEMs. The process starts by creating a universal draft model from the reactions and metabolites in the BiGG Models^159^, and enhancing it with manually annotated key aspects of bacterial metabolism to finalize the universal model. CarveMe then customizes this model for specific species using a process called ‘carving’, which includes gap-filling and removing irrelevant reactions and metabolites for each species^153^. This pipeline rapidly reconstructs metabolic models from genome sequences while maintaining critical metabolic functions.

Gapseq, another automated tool for model reconstruction, utilizes multiple biochemical databases to predict pathways from genetic content. In contrast to other tools, its reaction database sources from UniProt^160^ protein sequence database and the Transporter Classification Database (TCDB)^161^, encompassing 131,207 unique sequences^154^. These sequences contribute to 15,150 reactions and 8,446 metabolites, which are integrated into the universal model for reconstruction and gap-filling, thus providing a comprehensive approach to model building.

The primary technique for inferring the phenotypic behavior of organisms from GEMs is constraint-based reconstruction and analysis (COBRA)^162,163^. COBRA employs a systems biology approach to model an organism’s phenotypic behavior mathematically and computationally under various constraints. These constraints can represent genetic variations, environmental conditions, or interactions between different behaviors. Within the COBRA framework, flux balance analysis (FBA) stands out as a widely recognized method. FBA uses mathematical techniques to solve linear problems and determine the optimal metabolic fluxes (either mass or rate) within a reconstructed metabolic model under specific constraints^164^. FBA is particularly useful for simulating various biological phenomena, including maximal growth rates, the rates of metabolite production, and the impact of gene knockouts. These COBRA methods are accessible through a range of open-source software packages^165–168^, with the COBRA toolbox being the most popular.

GEMs for human gut bacteria are frequently utilized to predict metabolic interactions between microbes and conduct community metabolic modeling. Tools such as CASINO^169^, BacArena^170^ and the Microbiome Modeling Toolbox^163^ are among the most popular for these purposes. Many bacterial species in the gut depend metabolically on other species, and this dependency often dictates their co-occurrence within microbial communities^171^. Modeling these metabolic interactions is key to understanding the structure and resilience of microbial ecosystems, including the human gut microbiome. Moreover, metabolic modeling of a personal gut microbiome can uncover the roles of specific metabolites in human diseases. This aspect of modeling is particularly important because it can reveal connections between the microbiome, diseases, and potential treatments^172–174^. Additionally, modeling interactions between the host, microbiome, and diet can inform personalized dietary recommendations or drug dosages^175^. Hence, community-wide metabolic modeling that leverages both MAGs and GEMs is poised to make significant contributions to precision microbiome medicine^176^.

## Limitations and challenges

There are challenges and limitations in the current application of genome-resolved metagenomics within human microbiome research. First, a significant proportion of MAGs within existing databases represent incomplete genomes. These genomes often contain gaps. The quality of reference genomes plays a crucial role in the success of subsequent genome-centric microbiome analyses. Therefore, active research is underway to develop methods capable of reconstructing complete MAGs (cMAGs) without any gaps^48^. Traditional short-read sequencing techniques fall short when it comes to assembling highly conserved sequence regions across different species, such as 16S rRNA genes, and are unable to capture genomic regions transferred between species through HGT. Researchers are exploring methods that incorporate long-read sequencing as a solution. Initially, hybrid sequencing, which combines the nucleotide-level precision of short-read sequencing with the template of long-read sequences, was employed to construct cMAGs^177^. More recently, employing only high-fidelity long-read sequencing has also proven successful in constructing cMAGs^178^. The use of high-fidelity long-read metagenomic sequencing is expected to lead to a rapid increase in the availability of complete genomes of commensal bacteria in the human body.

Second, the majority of MAGs have been assembled from metagenomic samples originating from a limited number of countries^179^. This unequal representation in microbiome data, and consequently in the assembled genomes, can lead to a variety of issues. These issues include an incomplete understanding of microbiome diversity across different populations and an incomplete cataloging of reference microbial genomes. Potential consequences include inconsistencies in identifying disease-associated microbes and misinterpretations in comparative microbiome studies. Therefore, future efforts in MAG-based human microbiome research should prioritize underrepresented populations.

Third, applying genome-resolved metagenomics to low-biomass samples, such as tissue microbiomes, is challenging. In these cases, only a small fraction of shotgun sequencing reads are derived from microbial genomes. To address this challenge, various methods for host DNA removal have been developed^180–183^ and currently several host DNA removal kits are commercially available^184,185^. Effectively enriching bacterial DNA significantly increases the likelihood of reconstructing MAGs from low-biomass samples. This extends the applicability of the genome-resolved metagenomics approach to a wider array of microbial communities within the human body.

### Supplementary information

Supplementary Table 1
