# Supplementary material for: Genome-resolved metagenomics: a game changer for microbiome medicine
Source: Exp Mol Med. 2024 Jul 1;56(7):1501–12. doi: 10.1038/s12276-024-01262-7 (PMC11297344; doi:10.1038/s12276-024-01262-7)
Supplement: Supplementary file 1 — Supplementary Table 1 [file 12276_2024_1262_MOESM1_ESM.pdf]

Supplementary Table 1. Approaches and tools for genome-resolved metagenomics

a. Approaches and tools for sequencing read assembly

Approach	Pros	Cons	Tools
**Single-assembly**	• Less likely to merge reads derived from closely related species in different samples	• Impossible to assemble reads from low-abundant species due to lack of sufficient reads for assembly	metaSPAdes[1]<br>MEGAHIT[2]
**Co-assembly**	• Possible to assemble reads from low-abundant species by collecting reads from multiple samples	• Requires high computational resources<br>• Likely to mix highly closely related strains<br>- It can assemble chimera contigs.<br>- Assembler can made fragmented contigs and miss the strain-specific details.	

b. Approaches for binning

Approach	Pros	Cons
**Single-coverage binning**	• Possible for parallel binning.<br>• Likely to cluster the strain-specific accessory genome, which is co-abundant only one sample.	• Likely to include contamination, which is co-abundant only one sample.
**Multi-coverage binning**	• Possible to obtain a co-abundant core genome shared across multiple samples.<br>• Reduce contamination, which is co-abundant only one sample.	• Impossible for parallel binning.<br><br>• Likely to miss strain-specific accessory genome.

c. Tools for binning

Binning tools	Features extraction	Category of ML	Clustering algorithm
AAMB[3]	Co-abundance + k-mer frequency (TNF)	Unsupervised	Deep learning (Autoencoder-AAE)
SemiBin2[4]	Co-abundance + k-mer frequency (TNF)	Self-supervised	Deep learning (Siamese neural networks)
MetaBinner[5]*	Co-abundance + k-mer frequency (TNF) + single-copy marker gene	Semi-supervised	K-means clustering
Binny[6]	Co-abundance + k-mer frequency (k=2,3,4) + single-copy marker gene	Semi-supervised	Density-based clustering (HDBSCAN)
SemiBin[7]	Co-abundance + k-mer frequency (TNF) + cannot link constraints	Semi-supervised	Deep learning (Siamese neural networks)
MetaDecoder[8]	Co-abundance + k-mer frequency (TNF) + single-copy marker gene	Semi-supervised	Probabilistic model (DPGMM + GMM)
VAMB[9]	Co-abundance + k-mer freqeuncy (TNF)	Unsupervised	Deep learning (Autoencoder-VAE)
MetaBAT2[10]	Co-abundance + k-mer frequency (TNF)	Unsupervised	Graph-based clustering (weighted graph)
Maxbin2[11]	Co-abundance + k-mer frequency (TNF) + single-copy marker gene	Unsupervised	Probabilistic model (EM)
CONCOCT[12]	Co-abundance + k-mer frequency (TNF)	Unsupervised	Probabilistic model (GMM)
* contains internal bin refinement function			

d. Tools for bin refinement

Tool	Ensemble approach	Scoring approach	Pros	Cons	Max. No. binning tools
DAStool[13]	Aggregation of bin sets clustered by different binning tools	Using 51 bacterial and 38 archaeal single copy marker genes.	• Fast due to scoring algorithm<br>• Improves the completeness of final bin due to aggregation	• Susceptible to contamination due to aggregation<br>• Quality scoring based limited bacterial or archaeal marker genes	No limit
metaWRAP[14]	Merging hybrid bin sets using overlap	Using lineage-specific single copy marker genes (checkM)	• Quality scoring based on more diverse lineage-specific marker<br>• Improves the contamination of bin because hybrid bins are	• Slow due to scoring algorithm - quality assessment by checkM is carried out for every original and hybrid bin<br>• Can loses completeness	3

			made by overlapped contig	due to overlap	
MAGScoT[15]	Merging hybrid bin sets using overlap	Using 120 bacterial and 53 archaeal single copy marker genes	• Fast due to scoring algorithm	• Quality scoring based limited bacterial or archaeal marker genes	No limit

e. Tools for bin quality assessment

Tool	Completeness	Contamination	Approach	Pros	Cons
checkM[16]	O	O	Using lineage-specific marker gene set	• Accurate for known lineages	• Inaccurate for novel lineages that lack of marker gene sets and small species that lack universal marker genes
checkM2[17]	O	O	Using every gene + ML	• Evaluate novel lineages	• Incorrect assessment of taxonomically distant derived contamination, such as domain-level
GUNC[18]	X	O	Using every gene + lineage homogeneity of genome		• Incorrect assessment of taxonomically close derived contamination

Supplementary References

1 Nurk, S., Meleshko, D., Korobeynikov, A. & Pevzner, P. A. metaSPAdes: a new versatile metagenomic assembler. *Genome Res* **27**, 824-834 (2017). https://doi.org:10.1101/gr.213959.116

2 Li, D., Liu, C. M., Luo, R., Sadakane, K. & Lam, T. W. MEGAHIT: an ultra-fast single-node solution for large and complex metagenomics assembly via succinct de Bruijn graph. *Bioinformatics* **31**, 1674-1676 (2015). https://doi.org:10.1093/bioinformatics/btv033

3 Lindez, P. P. *et al.* Adversarial and variational autoencoders improve metagenomic binning. *Commun Biol* **6**, 1073 (2023). https://doi.org:10.1038/s42003-023-05452-3

4 Pan, S., Zhao, X. M. & Coelho, L. P. SemiBin2: self-supervised contrastive learning leads to better MAGs for short- and long-read sequencing. *Bioinformatics* **39**, i21-i29 (2023). https://doi.org:10.1093/bioinformatics/btad209

5 Wang, Z., Huang, P., You, R., Sun, F. & Zhu, S. MetaBinner: a high-performance and stand-alone ensemble binning method to recover individual genomes from complex microbial communities. *Genome Biol* **24**, 1 (2023). https://doi.org:10.1186/s13059-022-02832-6

6 Hickl, O., Queiros, P., Wilmes, P., May, P. & Heintz-Buschart, A. binny: an automated binning algorithm to recover high-quality genomes from complex metagenomic datasets. *Brief Bioinform* **23** (2022). https://doi.org:10.1093/bib/bbac431

7 Pan, S., Zhu, C., Zhao, X. M. & Coelho, L. P. A deep siamese neural network improves metagenome-assembled genomes in microbiome datasets across different environments. *Nat Commun* **13**, 2326 (2022). https://doi.org:10.1038/s41467-022-29843-y

8 Liu, C. C. *et al.* MetaDecoder: a novel method for clustering metagenomic contigs. *Microbiome* **10**, 46 (2022). https://doi.org:10.1186/s40168-022-01237-8

9 Nissen, J. N. *et al.* Improved metagenome binning and assembly using deep variational autoencoders. *Nat Biotechnol* **39**, 555-560 (2021). https://doi.org:10.1038/s41587-020-00777-4

10 Kang, D. D. *et al.* MetaBAT 2: an adaptive binning algorithm for robust and efficient genome reconstruction from metagenome assemblies. *PeerJ* **7**, e7359 (2019). https://doi.org:10.7717/peerj.7359

11 Wu, Y. W., Simmons, B. A. & Singer, S. W. MaxBin 2.0: an automated binning algorithm to recover genomes from multiple metagenomic datasets. *Bioinformatics* **32**, 605-607 (2016). https://doi.org:10.1093/bioinformatics/btv638

12 Alneberg, J. *et al.* Binning metagenomic contigs by coverage and composition. *Nat Methods* **11**, 1144-1146 (2014). https://doi.org:10.1038/nmeth.3103

13 Sieber, C. M. K. *et al.* Recovery of genomes from metagenomes via a dereplication, aggregation and scoring strategy. *Nat Microbiol* **3**, 836-843 (2018). https://doi.org:10.1038/s41564-018-0171-1

14 Uritskiy, G. V., DiRuggiero, J. & Taylor, J. MetaWRAP-a flexible pipeline for genome-resolved metagenomic data analysis. *Microbiome* **6**, 158 (2018). https://doi.org:10.1186/s40168-018-0541-1

15 Ruhlemann, M. C., Wacker, E. M., Ellinghaus, D. & Franke, A. MAGScoT: a fast, lightweight and accurate bin-refinement tool. *Bioinformatics* **38**, 5430-5433 (2022). https://doi.org:10.1093/bioinformatics/btac694

16 Parks, D. H., Imelfort, M., Skennerton, C. T., Hugenholtz, P. & Tyson, G. W. CheckM: assessing the quality of microbial genomes recovered from isolates, single cells, and metagenomes. *Genome Res* **25**, 1043-1055 (2015). https://doi.org:10.1101/gr.186072.114

17 Chklovski, A., Parks, D. H., Woodcroft, B. J. & Tyson, G. W. CheckM2: a rapid, scalable and accurate tool for assessing microbial genome quality using machine learning. *Nat Methods* **20**, 1203-1212 (2023). https://doi.org:10.1038/s41592-023-01940-w

18 Orakov, A. *et al.* GUNC: detection of chimerism and contamination in prokaryotic genomes. *Genome Biol* **22**, 178 (2021). https://doi.org:10.1186/s13059-021-02393-0
